# Rethinking the Conditions and Mechanism for Glymphatic Clearance

**DOI:** 10.3389/fnins.2021.624690

**Published:** 2021-04-08

**Authors:** Craig F. Ferris

**Affiliations:** Department Psychology and Pharmaceutical Sciences, Center for Translational Neuroimaging, Northeastern University, Boston, MA, United States

**Keywords:** microvasculature, brain temperature, circadian rhythm, magnetic resonance imaging, diffusion weighted

## Abstract

Critical studies that form the foundation of the glymphatic system and the clearance of metabolic by-products of unwanted proteins from the brain are reviewed. Concerns are raised about studying glymphatic flow in anesthetized animals and making assumptions about the whole brain based upon data collected from a cranial window on the cortex. A new model is proposed arguing that the flow of cerebral spinal fluid and parenchymal clearance in the perivascular system of unwanted proteins is regulated by circadian changes in brain temperature and blood flow at the level of the microvasculature.

## Introduction

**FIGURE 1:**
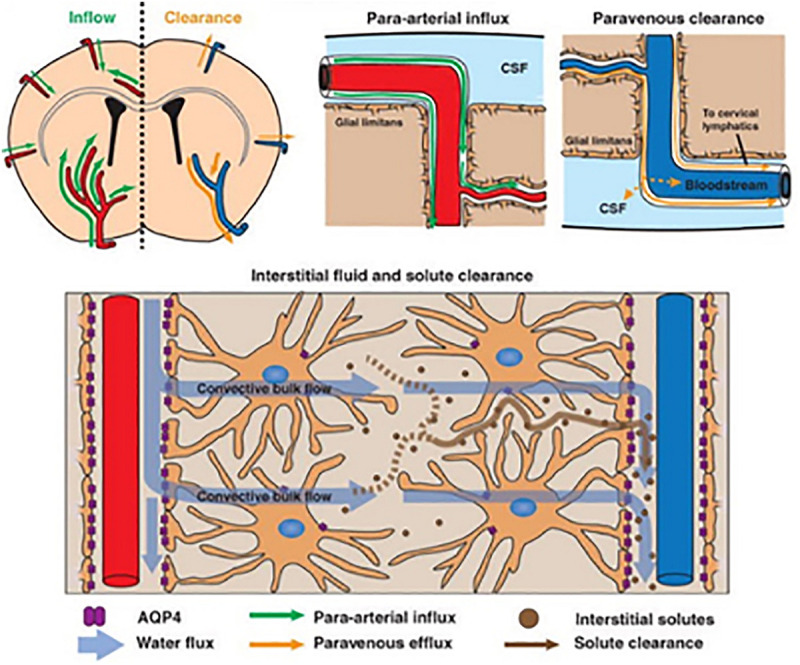
The schematic shows glymphatic clearance of solute from perivascular space of penetrating arteries (top right) across the brain parenchyma by convection into the perivascular space of penetrating veins. Adapted from Iliff et al. (2012).

The glymphatic system has been identified as playing a critical role in essentially all aspects of human health such as Alzheimer’s and Parkinson’s disease, aging, diabetes, stroke, head injury, glaucoma, and psychiatric disorders (Iliff et al., 2012; Xie et al., 2013; Wostyn et al., 2017; Kim et al., 2018; Rasmussen et al., 2018; Benveniste et al.,2019a,b; Piantino et al., 2019; Sundaram et al., 2019). The glymphatic system, by nature of its over 530 publications, has assumed the status of a scientific theory. Shown in Figure 1 is a schematic depicting the route of drainage of unwanted proteins from the brain (Iliff et al., 2012). Aided by aquaporin 4 (AQP4) water channels, cerebrospinal fluid (CSF) moves by convection from the perivascular space (PVS) of arteries through the extracellular space of the brain parenchyma to the PVS of veins for clearance (Iliff et al., 2012; Nedergaard, 2013). These vessels are typically depicted as penetrating arteries and veins along the surface of the brain emphasizing their large perivascular Virchow-Robins space. The original (Iliff et al., 2012; Nedergaard, 2013) and most recent promulgated models of glymphatic clearance do not include capillaries (Rasmussen et al., 2018; Braun and Iliff, 2020; Taoka and Naganawa, 2020); yet there is evidence of PVS around the microvasculature (Rennels et al., 1985; Bedussi et al., 2017; Pizzo et al., 2018).

Studies report that sleep is a necessary condition for proper glymphatic function such that unwanted proteins generated during the day with increased brain activity are cleared at night during sleep (Xie et al., 2013; Benveniste et al., 2019a; Holth et al., 2019). Indeed, the present paradigm argues disruption in sleep, a common problem in human health, is a contributing factor to numerous neurodegenerative diseases (Xie et al., 2013; Benveniste et al., 2019a). Mechanistically, the absence of a potentially expansive perivascular annular volume of the microcirculation and the requirement for sleep are difficult to explain. Why must the brain wait until sleep to clear something made in the day? Practitioners of the glymphatic theory argue clearance is restricted during waking hours because the extracellular space (ECS) is reduced by 60% during that time, essentially blocking convection from the artery to vein (Xie et al., 2013). Reducing the volume of the ECS by 60% across the brain during waking seems implausible for obvious reasons. The effects on transmembrane potentials caused by these dramatic changes in osmolarity and electrolyte concentrations are reported only in injured tissue (e.g., ischemia), and would probably be unlikely over the normal sleep-wake cycle (Sykova and Nicholson, 2008). These claims may be an artifact of experimental design e.g., studying glymphatic flow in anesthetized animals, making assumptions about the whole brain based upon data collected from a cranial window on the cortex, and limiting access to the PVS to a single route—the cisterna magna (CM). This perspective will discuss each of these issues. A new model is proposed arguing that flow of CSF and parenchymal clearance in the perivascular system of unwanted proteins is regulated by circadian changes in brain temperature and blood flow at the level of the microvasculature.

The whole premise of the glymphatic theory—the convection of metabolic by-products of unwanted proteins from the PVS of arteries to the PVS of veins—is founded on studies in mice focused on a small patch of superficial cerebral cortex 150 μm in depth (Iliff et al., 2012). This limited view of the brain is necessary because two photon imaging is used to follow movement of fluorescent labeled tracers along the PVS following injection into the CM. Imaging arteries at a chosen optical depth of 100–200 μm allows clear identification of tracer in the PVS—what have been described in human anatomy as Virchow-Robins spaces—and a modest amount of tracer beyond this limit described as influx into the parenchyma. Within the same optical image of approximately 200 μm in diameter, veins can be identified accumulating tracers in their PVS. This observation has led to the highly publicized drawings in research reports and reviews promoting the convection theory of glymphatic flow, as shown in Figure 1. Included in this model are the end feet of astrocytes that ensheath all the cerebral vasculature, arteries, capillaries, and veins (Papadopoulos and Verkman, 2013). Thus, the cerebral blood vessels are a tube-within-a-tube separated by the PVS. The surface area lining the astrocytic end feet have a high density of AQP4 water channels (Nielsen et al., 1997). These water channels have a key role in the glymphatic model, as they are proposed to be the conduit for convection (Iliff et al., 2012).

## The Confound of Anesthesia

**FIGURE 2:**
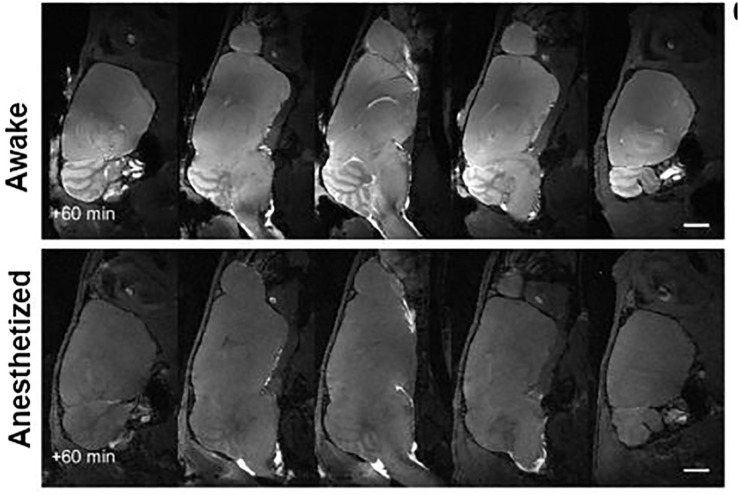
Gadolinium-enhanced magnetic resonance imaging of the mouse brain after injection of contrast agent into the cisterna magna under awake or isoflurane general anesthesia. Adapted from Gakuba et al. (2018).

The original publication in 2012 on glymphatic flow by Iliff, Nedergaard, and coworkers followed clearance of tracer injected into the lateral cerebroventricle (ICV) of anesthetized mice. This is an obvious choice and one used routinely today to study the distribution, clearance and activity of drugs and genetic therapeutics on the brain (Glascock et al., 2011). However, the authors reported the tracer did not move from the lateral ventricle. Only when tracer was injected into CM, directly into the subarachnoid space that surrounds the CNS, was there movement and penetrance down pial vessels. This observation is not surprising as these are essentially a continuous space (Zhang et al., 1990; Bedussi et al., 2017). In a subsequent study the Nedergaard group tried to repeat this in awake mice and reported no movement or penetrance of trace from CM injections (Xie et al., 2013). This led to the claim that CSF does not circulate in awake animals when introduced ICV or intrathecally (Iliff et al., 2012; Xie et al., 2013). There seems to be a misconception by practitioners of the glymphatic theory that molecules injected ICV or CM in awake animals do not circulate, penetrate the parenchyma, and clear. There are numerous studies dating back decades showing psychostimulants, peptides, and neurotransmitters have immediate effects—within minutes—on nociception, gastric motility, heart rate, blood pressure, urine volume, hormone secretion and behavior following ICV injection (for examples see Pedigo et al., 1975; Hall, 1981; Berkowitz and Sherman, 1982; Cowan et al., 1985; Tsukamura et al., 2000; Hanada et al., 2001; Stone et al., 2003; Barcia and Gallego, 2009; Onogi et al., 2010; Toll et al., 2012). Radiolabeled peptides spread across the parenchyma hitting their target receptors and peaking within 2–5 min after ICV injection and almost immediately appear in the systemic blood (Saija et al., 1989). What do all these studies have in common—the animals are awake. Brevard et al., reported on the genesis of pentylenetetrazol (PTZ)-induced generalized seizures in fully awake rats during MRI (Brevard et al., 2006). Within 5–10 s after ICV PTZ injection the anterior thalamus and retrosplenial cortex show significant increases in brain activity followed secs later by the hippocampus and by 30 s, seizure onset. In a recent study, Ma et al. (2019) followed efflux of fluorescent tracer following ICV injection in awake mice vs. anesthetized mice. Tracer rapidly exits the subarachnoid space through the lymphatic system to the systemic circulation in awake mice but not so in anesthetized mice. These researchers also noted that there was little accumulation of trace in the superficial layer of the cerebral hemispheres in the awake condition due to rapid clearance, but a high concentration of trace under anesthesia. This confinement of trace to the subarachnoid space under anesthesia favors the influx down PVS of penetrating arteries and veins. The most provocative finding by Ma and coworkers was that death enhances the influx of trace down the PVS in anesthetized mice. *In vivo* imaging in anesthetized mice given trace via the CM always show a high concentration in the subarachnoid space and superficial cerebral hemispheres but not the subcortical areas. It is only upon death and preparation of samples for histology is the trace reported in deeper layers of cortex and underlying parenchyma (Iliff et al., 2012). Gakuba et al. (2018) performed a series of elegant studies using near infrared fluorescence imaging and contrast enhanced MRI to characterize the distribution of tracers injected CM in awake mice and compared these results with anesthesia. As Figure 2 clearly shows, contrast agent spreads across the brain when mice are awake but is severely limited with anesthesia. One of the unique features of this study was scheduling the imaging session during the night when mice are normally active. It was reported by Cai et al. (2020) that ICV injection of MRI contrast agent in awake rats effectively moves everywhere in the brain with greater accumulation during the dark phase when rats are active indicating less clearance, a conditioned that may have enhanced influx in awake mice injected CM. The study by Ma et al., raises serious doubts about the claims of parenchymal influx and efflux using the anesthetized CM protocol. This is in stark contrast to studies of the Nedergaard group that movement of tracer occurs only under anesthesia or sleep and then down medium and large vessels with a modest accumulation in the surrounding parenchyma.

## Generalizations From a Small Window

The absence of glymphatic function in awake rats that causes a 95% reduction in convection from artery to vein—the foundation of the glymphatic theory—is explained by a 60% decrease in the ECS (Xie et al., 2013). This reduction in the extracellular compartment increases resistance and hinders convection. To account for the lost interstitial fluid the Nedergaard group proposed an adrenergic driven expansion of the parenchymal cell volume, i.e., water moves from the ECS to the intracellular compartment. The evidence for these claims come from the small cranial window over the cortex using the tetramethyl-ammonium (TMA) method in head-fixed mice to measure the ECS at a depth of 100–200 μm. The alpha (α) or ratio of extracellular volume over tissue volume is approximately 22% under anesthesia but drops to 13% if mice are aroused. A cocktail of adrenergic antagonists injected into the CM in awake mice produces the increase in this α shown with anesthesia. These dramatic, acute changes in the α are not seen under normal physiological conditions (Sykova and Nicholson, 2008) but are reported in the rodent spinal cord in response to electrical, chemical, or thermal injury (Svoboda and Sykova, 1991), hippocampus in models of epilepsy (Arranz et al., 2014) and neocortex in response to large changes in osmolarity (Kume-Kick et al., 2002).

It is assumed, but never explicitly stated in the glymphatic theory that the conditions and mechanisms for artery to vein convection is global. That is to say, the movement of tracer observed with two photon imaging and the change in the α with the TMA method occur everywhere. This notion of increasing and decreasing the ECS by 60% as one transitions between sleep/anesthesia and arousal was challenged by Gakuba et al. (2018) using the apparent diffusion coefficient (ADC) in diffusion weighted imaging as a proxy for the ECS, i.e., an increase ECS would result in an increase in ADC and vice versa. They imaged mice transitioning from fully awake to anesthetized and found no significant differences in ADC values in the brain. Studies performed at the National Institutes of Health following changes in ADC values in awake and sleeping volunteers reported no differences in global ADC values or major brain areas e.g., thalamus, caudate/putamen, amygdala, accumbens, and hippocampus (Demiral et al., 2019). The proposition by the Nedergaard group that sleep, or anesthesia will enhance clearance via a mechanism of dramatically increasing the ECS should therefore be rejected based on this scientific evidence alone, leaving aside the argument that it is highly improbable based on the normal physiology of the brain. Hence the condition—sleep and anesthesia, and mechanism—expansion of the ECS, appear to be an artifact of experimental design.

## Where Are the Capillaries?

The organizational model of the glymphatic system as shown in Figure 1 does not include capillaries. This is odd, since the microstructure of the neurovascular unit providing rapid exchange between capillaries and neurons is an axiom of neuroscience. While the PVS around large and medium penetrating blood vessels is easily observed by *in vivo* imaging and postmortem histology (Iliff et al., 2012; Brinker et al., 2014) the evidence for PVS or peri-capillary space around microvessels still requires firm evidence. Techniques for imaging an annular or paravascular space for convection along capillaries *in vivo* do not exist. Tissue preparation for electron microscopy can introduce structural artifacts. Nonetheless, Bedussi et al. (2017) used correlative light electron microscopy to show CM injected trace along capillary endothelial cells. Pizzo et al. (2018) reported CM injected antibodies localized around putative capillaries using immunofluorescent confocal microscopy. These researchers also proposed that the PVS is continuous, providing CSF movement from arteries to capillaries to veins. Earlier, Rennels et al. (1985) had made a similar suggestion based on ICV injections of horseradish peroxidase in anesthetized cats and dogs, showing perfuse enzyme staining around parenchymal capillaries 4–6 min after injection. This concept of CSF access through a continuous PVS across the vascular tree attends more closely to the importance of the neurovascular unit in clearing metabolic waste from the surrounding parenchyma as opposed to the glymphatic theory of convection from arteries to veins. For a comprehensive review on the organization of the perivascular space see Bowling et al. (1993). While it easy to dismiss something because it does not seem right, perhaps this concern can be argued in another way. Why would evolution assign the critical role of waste removal from the brain to large blood vessels and their annular volume that represent only a small fraction of the total vascular surface area for exchange?

## Summary

This perspective does not dispute the findings in animals that the brain clears itself of unwanted proteins through the perivascular system and meningeal lymphatics. This perspective does not dispute the fact that this clearance is greatest at night during rest and sleep. What is contested are the conditions and mechanisms responsible for this clearance and the interpretations/generalizations that follow. Much of the glymphatic theory is predicated on observations in anesthetized mice taken from penetrating arteries and veins in the cortex following CM injection of trace. It is assumed that what happens in this small cranial window to the movement of trace across the PVS is global, but this has not been tested. The argument for convection over diffusion (Asgari et al., 2016; Abbott et al., 2018; Pizzo et al., 2018) in the glymphatic theory is based in part on the dramatic artificial increase in extracellular space with sleep and anesthesia and the role of AQP4, and as such, must be reconsidered. These points of concern are not new to this perspective but have been raised by other researchers (Brinker et al., 2014; Hladky and Barrand, 2014; Bakker et al., 2016; Bedussi et al., 2017; Abbott et al., 2018; Gakuba et al., 2018; Smith and Verkman, 2018; Ma et al., 2019).

## A New Hypothetical Model for Brain Clearance of Metabolic Waste

**FIGURE 3:**
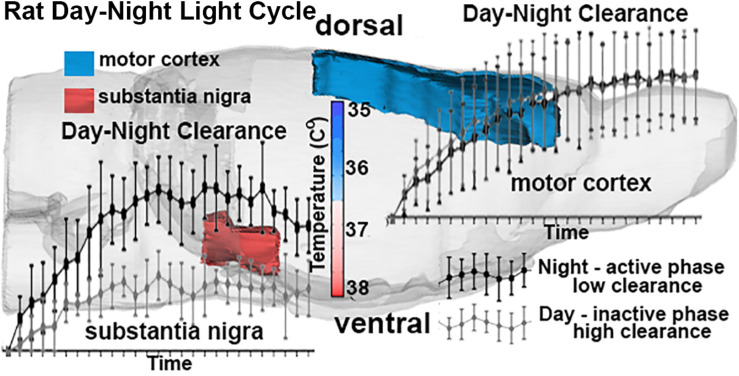
The schematic depicts the dorsal/ventral gradient of brain temperature with clearance curves in the motor cortex (blue) and SN (red) following injection of ICV contrast in awake rats. The data was collected repeatedly over 90 min post injection into rats scanned at night when active and day when resting. All rats were imaged awake regardless of day-night cycle—none were sleeping during the imaging session. Adapted from Cai et al. (2020).

The brain utilizes more glucose and consumes more oxygen than any other organ in the body and generates an enormous amount of heat given its size relative to the total body mass. Brain temperatures are not a simple reflection of core body temperature; instead, they are higher than the body temperature and heterogeneous (Zhu et al., 2009; Mrozek et al., 2012; Wang et al., 2014). Intracerebral temperatures vary across the dorsal–ventral axis as depicted in Figure 3. Brain temperatures are lowest in the cortex and highest in the midbrain core and ventral surface of the brain (Wang et al., 2014). The lower temperatures along the dorsal surface of the brain are due to greater radiation and convection of heat through the large surface of the cerebrum and relatively thin overlying skull and skin. The somatosensory and motor cortices of the cerebrum do not show a significant diurnal variation in the clearance of contrast agent (CA) as compared to more ventral areas like ventral midbrain substantia nigra (SN) (Cai et al., 2020) as shown in Figure 3. Cai et al. (2020) were the first to show that perivascular clearance in awake rats was circadian, highest during the light phase and lowest during the dark phase, independent of sleep. These observations raised the possibility that the temperature of the brain has a significant effect on clearance.

**FIGURE 4:**
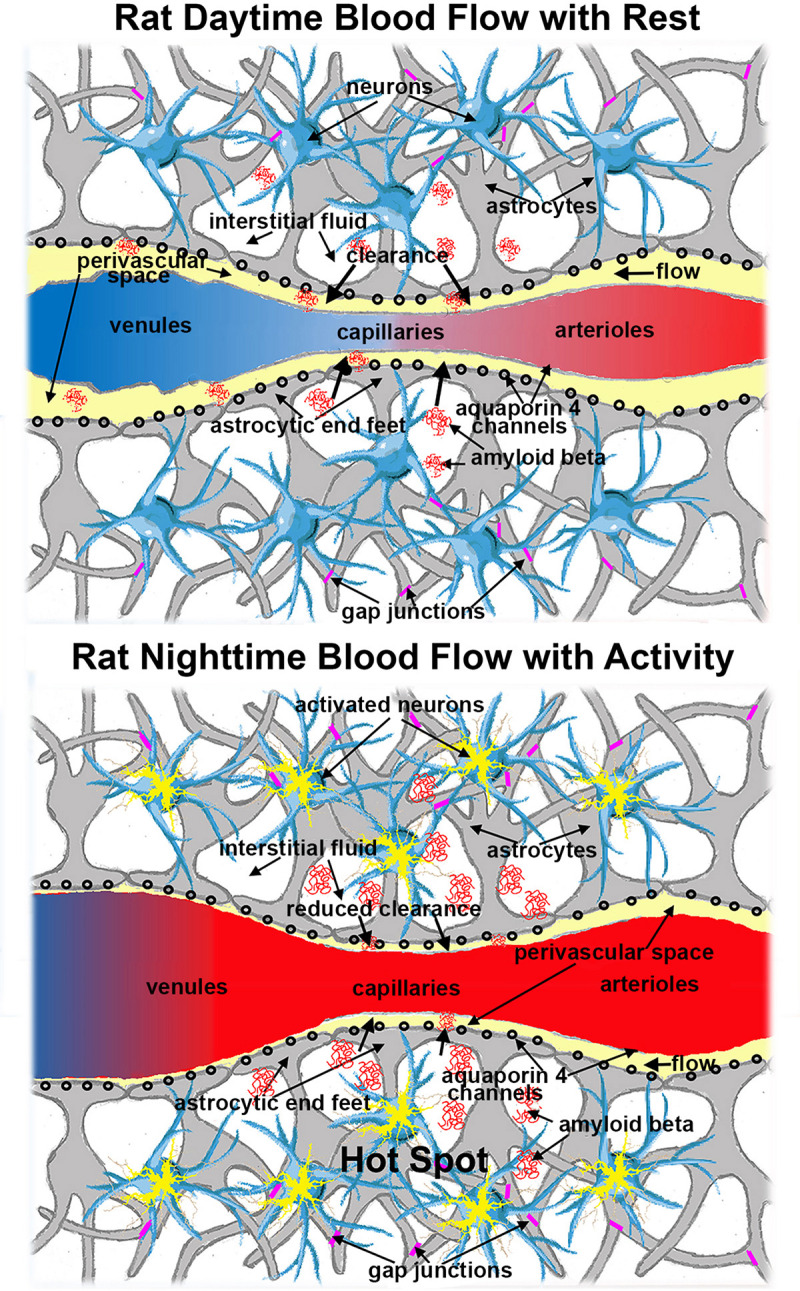
Schematic showing the expanded PVS (yellow) in the microcirculation during the daytime (inactive phase) as compared to the reduced PVS during the night (active phase). Red denotes oxygenated hemoglobin and blue deoxygenated hemoglobin. The increased cerebral blood volume (CBV) in areas of brain activity is the mechanistic foundation of BOLD imaging and functional imaging with CBV.

The brain serves as a heat source and the blood as a heat sink (Zhu et al., 2009; Mrozek et al., 2012; Wang et al., 2014). *In anesthetized animals this is reversed, the brain is colder than the blood* (LaManna et al., 1988; Erickson and Lanier, 2003; Zhu et al., 2009; Mrozek et al., 2012; Wang et al., 2014). This necessitates the study of awake animals free of anesthesia, a confound not sufficiently addressed in the glymphatic literature as the discussions have centered around what is the best anesthetic to use (Hablitz et al., 2019), and not that it should be avoided. Although it should be noted that different anesthetics may have different effects on brain temperature. Brain temperature is a circadian rhythm entrained by the light-dark (L-D) cycle (Refinetti and Menaker, 1992; Scheer et al., 2005). The diurnal change in temperature sets into motion numerous other biological rhythms including sleep (Buhr et al., 2010; Morf and Schibler, 2013). Changes in body temperature rise before awaking and lower before sleep (Van Someren, 2006). There is a circadian rhythm in metabolic heat production that is independent of sleep (Krauchi and Wirz-Justice, 1994). This anticipatory thermoregulation is controlled by the suprachiasmatic nucleus (SCN) of the hypothalamus (Honma, 2018). The temperature of the brain varies over the sleep–wake cycle of freely moving rats (Abrams and Hammel, 1965). Rats are nocturnal, so it is highest while rats are awake and active (dark) and lowest during sleep (light). Site specific brain activation causes an immediate increase in localized brain temperature, creating a thermal gradient between the parenchyma and surrounding microvasculature (Kiyatkin, 2019). The enhanced blood flow during the awake, active phase of the L-D cycle is necessary for moving blood gases and nutrients across the interstitial fluid/capillary interface and buffering temperature. *It is hypothesized in this perspective, that the increased cerebral blood volume and transluminal pressure would reduce or collapses the PVS of the capillaries and venules as depicted in* Figure 4. The venules but not the arterioles in Figure 4 are depicted as high compliance vessels readily distended by an increase in blood volume. This expected increase in blood volume to areas of high metabolic activity is the foundation of functional imaging using contrast enhanced cerebral blood volume fMRI or BOLD (blood oxygenation level dependent) fMRI (Buxton et al., 1998; Mandeville et al., 1999). Thus, the movement of non-permeable metabolites and waste products like amyloid-β and α-synuclein is restricted during this period. Regulation of brain temperature takes precedence over clearance.

I have argued that the ebb and flow of perivascular fluid over the circadian cycle is primarily influenced by temperature and blood flow. However, Fultz et al. (2019) correlated cycles of increased CSF flow in the cerebral ventricles (ca 1 every 20 s) to changes in EEG activity and BOLD signal in the cortex during NREM sleep. With the increase in EEG came the expected increase in BOLD, a surrogate measure of increased blood flow. As the BOLD signal receded the CSF flow increased. These results would fit the model shown in Figure 4 as the increased blood flow would impede movement of perivascular fluid along capillaries and venules. This finding introduces another condition and mechanism for perivascular clearance in addition to regulation of brain temperature as hypothesized here.

While purely speculative, the electrical activity alone, independent of blood flow, may play a role in promoting efflux. Consider astrocytes within a neurovascular unit as a hydrolytic syncytium, coupled through gap junctions (see Figure 4). Diffusion functional MRI shows that neural activity is associated with astrocytic and neuronal swelling (Andrew and MacVicar, 1994; Darquie et al., 2001; MacVicar et al., 2002; Le Bihan et al., 2006; Fields, 2011), an effect that can occur in the absence of an increase in BOLD (Abe et al., 2017). The flux of water across AQP4 channels in astrocytic endfeet is bidirectional (Amiry-Moghaddam et al., 2003). The activity driven, swelling of astrocytes would increase the intracellular hydrostatic pressure promoting efflux of water, solute, and unwanted proteins toward the capillary PVS. This *convection* would be realized across the neurovascular unit.

If the circulation of CSF thought perivascular system contributes to the removal of unwanted proteins that increase risk for Alzheimer’s and Parkinson’s disease, what can be done to enhance this process? To date there is no evidence of glymphatic clearance of amyloid-β and α-synuclein in humans (Smith and Verkman, 2018). Practitioners of the glymphatic theory argue that anesthesia or hypnotics that mimic sleep could increase clearance although this has not been shown (Benveniste et al., 2019a). The new model of clearance presented in this perspective based on circadian variation in brain temperature and blood flow would suggest this approach may not be fruitful. Instead, the most effect approach may be adhering to rest, reducing activity, and sleeping during the night and early morning hours, acknowledging the importance of circadian physiology—“don’t fight the clock.” A more speculative and therapeutic approach would be brain cooling—non-invasively stabilizing temperature at the circadian nadir of ca 36°C as recorded during human sleep (Waterhouse et al., 2012). The full potential for clearance is only realized after the need to regulate temperature is satisfied.

## Data Availability Statement

The original contributions presented in the study are included in the article/supplementary material, further inquiries can be directed to the corresponding author/s.

## Author Contributions

CF was responsible for writing this review.

## Conflict of Interest

CF has a financial interest in Animal Imaging Research, the company that manufactures technology for awake imaging.
